# Inhibition of SOS Response by Nitric Oxide Donors in *Escherichia coli* Blocks Toxin Production and Hypermutation

**DOI:** 10.3389/fcimb.2021.798136

**Published:** 2021-12-22

**Authors:** John K. Crane, Sarah R. Burke, Cassandra L. Alvarado

**Affiliations:** Department of Medicine, Jacobs School of Medicine and Biomedical Sciences, University at Buffalo, Buffalo, NY, United States

**Keywords:** zinc, Shiga toxins, nitrosothiols, enterohemorrhagic *E coli*, nitric oxide synthetase

## Abstract

**Background:**

Previous reports have differed as to whether nitric oxide inhibits or stimulates the SOS response, a bacterial stress response that is often triggered by DNA damage. The SOS response is an important regulator of production of Shiga toxins (Stx) in Shiga-toxigenic *E. coli* (STEC). In addition, the SOS response is accompanied by hypermutation, which can lead to *de novo* emergence of antibiotic resistance. We studied these effects *in vitro* as well as *in vivo*.

**Results:**

Nitric oxide donors inhibited induction of the SOS response by classical inducers such as mitomycin C, ciprofloxacin, and zidovudine, as measured by assays for *E. coli* RecA. Nitric oxide donors also inhibited Stx toxin protein production as well as *stx2* RNA *in vitro* and *in vivo*. *In vivo* experiments were performed with ligated ileal segments in the rabbit using a 20 h infection. The NO donor S-nitroso-acetylpenicillamine (SNAP) reduced hypermutation *in vitro* and *in vivo*, as measured by emergence of rifampin resistance. SNAP blocked the ability of the RecA protein to bind to single-stranded DNA in an electrophoretic mobility shift assay (EMSA) *in vitro*, an early event in the SOS response. The inhibitory effects of SNAP were additive with those of zinc acetate.

**Conclusions:**

Nitric oxide donors blocked the initiation step of the SOS response. Downstream effects of this blockade included inhibition of Stx production and of hypermutation. Infection of rabbit loops with STEC resulted in a downregulation, rather than stimulation, of nitric oxide host defenses at 20 h of infection.

## Introduction

Our laboratory became interested in the SOS response because of its importance in regulating the production of Shiga toxins in Shiga toxigenic E. coli (STEC) (Kimmitt et al., 2000), as well as its role as a generator of antibiotic resistance (Blázquez et al., 2018). Several years ago, Vareille et al. reported that nitric oxide donors inhibited the production of Shiga toxins from STEC by blocking the SOS pathway (Vareille et al., 2007). That study suggested that nitric oxide donors inhibited the SOS pathway in general, and not just Shiga toxin production. Other authors have reported that nitric oxide donors or their derivatives, however, might actually induce the SOS response rather than inhibiting it (Stupakova et al., 2000; Motohashi and Saito, 2002; Kim et al., 2005). Ichimura et al. reported that nitric oxide donors could induce Stx production *in vitro*, but only under anaerobic conditions {Ichimura, 2017 #6644}. Therefore, the question of whether NO is helpful or hurtful in STEC infection seemed important to resolve {Naïli, 2020 #6523}. We therefore initiated this study to determine if nitric oxide donors inhibited or stimulated the SOS response and if they affected production of Shiga toxins in particular. Last, we examined if nitric oxide donors would affect the hypermutator phenotype, which can trigger *de novo* emergence of antibiotic resistance (Cirz and Romesberg, 2006; Händel et al., 2015; Goodman, 2016; Bunnell et al., 2017; Blázquez et al., 2018).

The bacterial protein RecA is the primary sensor of DNA damage, because long stretches of single-stranded DNA are the trigger for initiation of the SOS response. RecA then induces its signaling partner, the inhibitor LexA, to cleave itself, allowing expression of the suite of genes involved in the SOS response. The genes for Stx1 and Stx2 are encoded within bacteriophage that parasitize STEC bacteria. Induction of the SOS response triggers the bacteriophage to exit from latency and begin the lytic phase of their life cycle, producing live, infectious phage particles that can infect other bacteria. Since RecA expression is an early and reliable marker of the SOS response, we began by measuring RecA expression using a *recA-lacZ* reporter *E. coli* strain (Mellies et al., 2007). We next measured *recA* expression by qRT-PCR, and we also measured production of Shiga toxin protein as well as expression of *stx2* RNA by qRT-PCR. In addition, we examined whether nitric oxide donors could inhibit hypermutation triggered by SOS-inducing drugs *in vitro* or *in vivo*. Last, we also examined if nitric oxide donors could block the ability of the RecA protein to recognize and bind to ssDNA using a fluorescent oligonucleotide probe.

## Results

**Figure 1:**
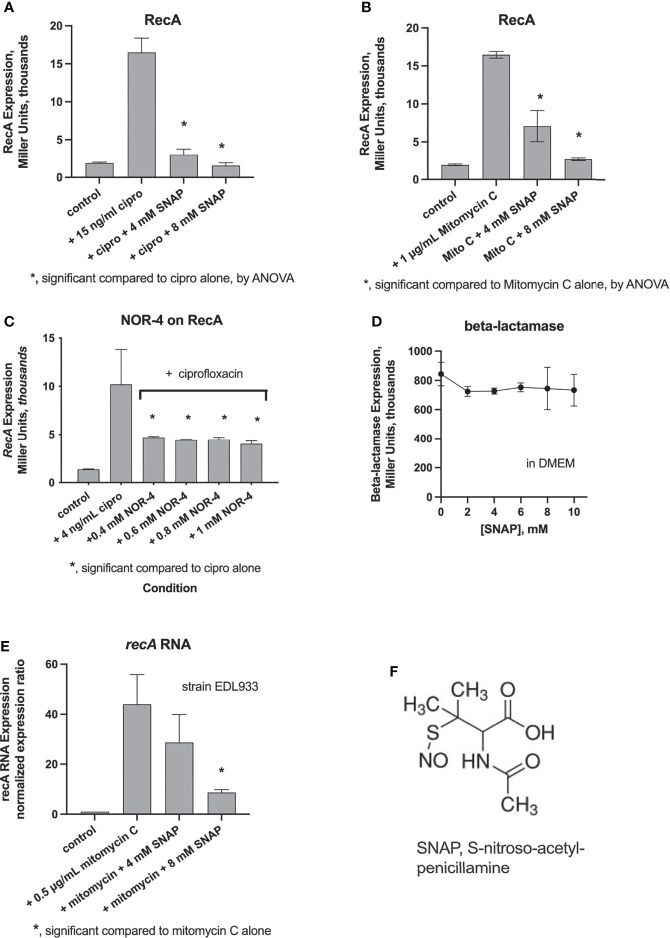
Panels **(A-C)**, measurement of induction of RecA using the *recA-lacZ* reporter strain, JLM281, by the Miller assay, as described in the *Methods* section. SNAP inhibited RecA expression triggered by ciprofloxacin **(A)** and mitomycin C **(B)**. The NO donor NOR-4 also inhibited ciprofloxacin-induced RecA expression **(C)**. Panel **(D)**, lack of inhibition by SNAP of expression of ß-lactamase in the negative control reporter, strain MCamp. **(E)**, inhibition of mitomycin-induced *recA* RNA abundance in STEC strain EDL933, by qRT-PCR. **(F)**, chemical structure of SNAP.

We measured RecA expression in response to various SOS-inducing stimuli and tested if nitric oxide donors would inhibit this induction, as reported by Vareille (Vareille et al., 2007). **Figure 1**, Panels A and B, show that the NO donor SNAP inhibited RecA expression triggered by ciprofloxacin and mitomycin C. We also tested the NO donor sodium nitroprusside and found that it was also effective in inhibiting SOS induction, although it was slightly less potent, with 8 mM nitroprusside required to observe inhibition similar to that of 4 mM SNAP (data not shown).

We also tested the NO donor NOR-4, the agent used by Vareille and colleagues, and found that NOR-4 inhibited ciprofloxacin-induced RecA expression. NOR-4, however, showed a plateau in its ability to inhibit RecA, which could not be overcome by increasing the concentration of the NO donor. As an additional control, we tested the effect of NO donors on a different reporter gene, in which ß-lactamase is fused to the same ß-galactosidase reporter (*bla-lacZ*) as used for RecA. **Figure 1D** shows that SNAP did not inhibit ß-lactamase expression in the negative control reporter.

Next, we measured the expression of *recA* RNA using reverse transcription and qRT-PCR. **Figure 1E** shows that mitomycin C again strongly induced *recA* expression, and this induction was almost completely reversed by SNAP. **Figure 1F** shows the chemical structure of SNAP.

**Figure 2:**
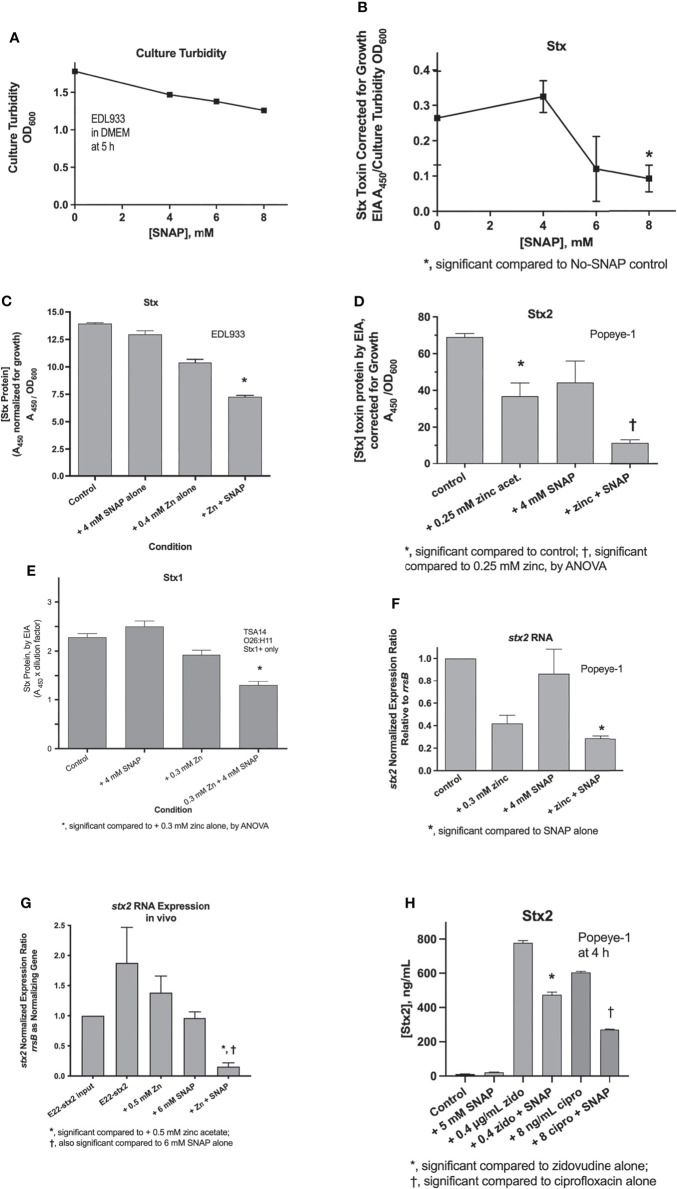
Effects of SNAP on Stx toxin protein and *stx* RNA expression. Panel **(A)**, modest inhibitory effects of SNAP on *E. coli* growth. Panels **(B–D)**, effect of SNAP on basal, i.e., unstimulated Stx toxin in STEC strains EDL933 and Popeye-1. Panel **(E)**, effect of zinc and SNAP on stx2 RNA in strain Popeye-1 *in vitro*. Panel **(F)**, effect of zinc and SNAP on stx2 RNA in rabbit STEC strain E22-stx2 *in vivo* in rabbit ileal loops at 20 h. Panels **(D–G)** show the additive inhibitory effects of zinc acetate and SNAP. Panel **(H)**, inhibition by SNAP of Stx2 toxin induced by zidovudine (zido) and ciprofloxacin (cipro).

We next assessed whether nitric oxide donors would inhibit the production of Shiga toxins (Stx) from various STEC strains *in vitro* and *in vivo* in the rabbit intestinal loop model. As a point of reference, **Figure 2A** shows that SNAP has only modest ability to inhibit bacterial growth, with 8 mM SNAP inhibiting growth of STEC EDL933 by 30%. In contrast, SNAP was quite active in its ability to inhibit Stx from the same strain, even when toxin production is normalized for the modest inhibition of growth. Since we had previously shown that zinc acetate inhibits Stx production (Crane et al., 2014), we also tested if the NO donor would show any additive effect in the presence of zinc. **Figure 2C** shows that SNAP did produce additive inhibition when added with submaximal concentrations of zinc in strain EDL933. We repeated this experiment using a different STEC strain, Popeye-1, which only produces Stx2. **Figure 2D** shows that zinc and SNAP also showed additive inhibitory activity against Stx2 production in Popeye-1. The combination of SNAP plus zinc also inhibited Stx in a strain, TSA14, that only produces Stx1 (**Figure 2E**). We next measured whether SNAP would inhibit expression of *stx2* RNA *in vitro* and *in vivo*. **Figures 2F, G** show that SNAP again produced additive inhibition when added with zinc *in vitro* in STEC. The effects of zinc and SNAP on *stx* RNA in **Figures 2F, G** show that SNAP reduces RNA abundance. Therefore, the beneficial effects of zinc and SNAP cannot be ascribed solely to effects on the bacterial envelope, such by trapping of Stx toxin protein in the periplasmic space. **Figure 2G** shows the effects of SNAP and zinc on *stx2* RNA *in vivo* in rabbit loops infected with STEC strain E22-stx2. **Figure 2H** shows that SNAP was able to inhibit zidovudine-induced and ciprofloxacin-induced Stx2 toxin protein production *in vitro*. The results of **Figure 2** showed that SNAP inhibited Stx toxin protein release, and *stx* RNA. The effects of NO were additive with zinc acetate, and were observed without and with inducers of the SOS response.

**Figure 3:**
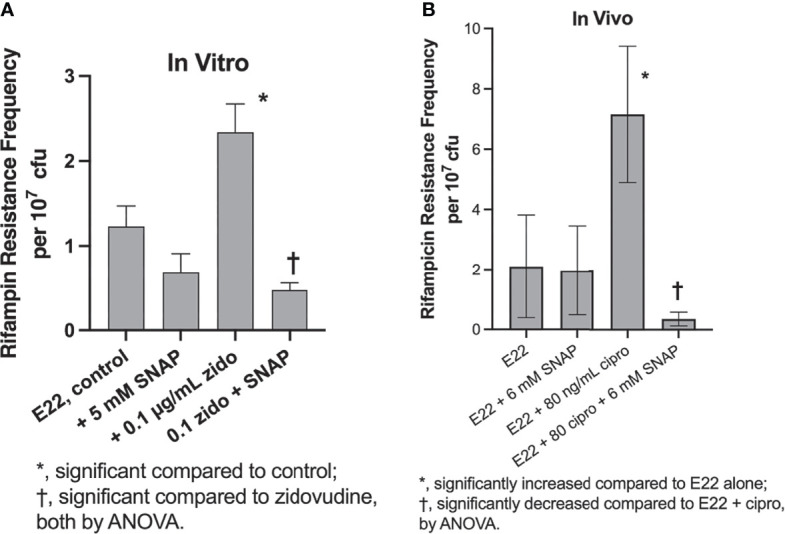
Inducers and inhibitors of hypermutation in rabbit EPEC strain E22 *in vitro* and *in vivo*. Panel **(A)**, effect of zidovudine and SNAP on rifampin resistance frequency in strain E22. Addition of zidovudine and SNAP was delayed until 1 h after the initiation of the subculture, and bacteria were collected at 4 h, and plated on plates of LB + 24 µg/mL rifampin, which is 4 X the rifampin MIC of the E22 parental strain. Panel **(B)**, inhibition by SNAP of ciprofloxacin-induced hypermutation *in vivo* in rabbit ileal segments, or “loops,” at 20 h of infection. Two loops were used for each infection condition shown, and each sample of loop fluid was plated in quadruplicate on LB + rifampin as well as on plain LB, the latter to determine total bacterial counts.

In addition to inducing Stx production, SOS inducers also trigger hypermutation in *E. coli* and other bacteria (Händel et al., 2015; Song et al., 2016; Crane et al., 2021). We tested if SNAP would also inhibit the hypermutation response in *E. coli* strain E22. **Figure 3A** shows that SNAP inhibited the zidovudine-induced increase in rifampin resistance frequency *in vitro* in strain E22. We also tested if SNAP would inhibit this hypermutation response *in vivo*. Based on our trial-and-error experience in the rabbit model (Crane et al., 2021), we found that higher concentrations of inducers were generally needed to observe a hypermutation response *in vivo* than *in vitro*. **Figure 3B** shows that 6 mM SNAP did inhibit ciprofloxacin-induced hypermutation *in vivo*, in a manner similar to the inhibitory effects of zinc *in vivo* (Crane et al., 2021).

**Figure 4:**
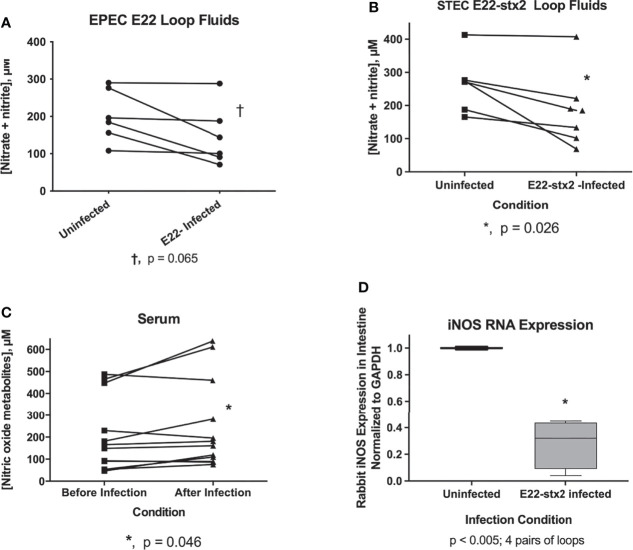
Evaluation of host nitric oxide defenses against rabbit EPEC and rabbit STEC *in vivo*. Panels **(A–C)**, nitric oxide metabolites, nitrate plus nitrite, were measured using a nitric oxide assay kit. Panel **(A)**, pairs of measurements showing nitric oxide metabolites in uninfected loops compared with E22-infected loops in the same animal. Results from six separate rabbit experiments are shown. A trend toward decreased nitric oxide metabolites in the infected loops did not reach statistical significance. Panel **(B)**, pairwise comparison of NO metabolites in loop fluid recovered from uninfected vs. E22-stx2 loops in 7 rabbit experiments. The decrease in NO metabolites reached statistical significance with the rabbit STEC strain. Panel **(C)**, NO metabolites in the serum of rabbit before and after infection with rabbit E.coli pathogens. Sera from rabbits infected with strain E22 and E22-stx2 were combined in this graph (n= 12 pairs). A very small increase in NO metabolites was noted in the serum obtained after infection compared to before infection. Panel **(D)**, expression of RNA encoding rabbit iNOS was measured in full thickness biopsies of intestinal tissue in 4 pairs of samples, from uninfected vs. E22-stx2-infected rabbit ileal loops. RNA abundance was normalized to glyceraldehyde phosphate dehydrogenase (GAPDH) for each condition, and the expression in infected loops was expressed compared to the control loop, using the 2^-ΔΔCt^ method, yielding 4 pairs of measurements from 4 rabbits.

Given the strong inhibition by NO donors of RecA and Stx, we wondered if the host intestinal tract was capable of generating NO as a host defense against EPEC or STEC. A previous report using cultured intestinal cells suggested that an effective nitric oxide host defense might not occur (Maresca et al., 2005). We measured nitric oxide metabolites *in vivo* in the intestinal loop fluids from rabbits loop infected with rabbit EPEC, strain E22, or rabbit STEC, E22-stx2. **Figure 4A** shows the nitric oxide metabolites in loops infected with strain E22 in comparison with uninfected control in the same animal. NO metabolites appeared to decrease in 3 of the 6 animals tested, but this decrease did not reach statistical significance. **Figure 4B** shows a similar experiment measuring NO metabolites in rabbit loops infected with STEC E22-stx2. With E22-stx2, NO metabolites decreased in the infected loops compared to the uninfected loops in the same animal, and this decrease was significant by paired t-test. In **Figure 4C**, we tested for NO metabolites in the serum of rabbits before and after a 20 h infection. Sera from E22-infected and E22-stx2-infected rabbits were combined in **Figure 4C**. In contrast to the intestinal loop fluids, NO metabolites rose slightly in serum after 20 h. Last, we measured inducible nitric oxide synthetase (iNOS) RNA expression in transmural intestinal biopsies by reverse transcription and qRT-PCR. Inducible NOS seemed to be the most relevant form of NOS to examine in the setting of intestinal infection, rather than endothelial NOS (eNOS) or neuronal NOS (nNOS). Again, we compared loops infected with E22-stx2 with the iNOS expression in the uninfected loop in the same animal. The results of **Figure 4D** showed that iNOS RNA expression decreased in the loops infected with the rabbit STEC strain to a level 28% of that in the control. **Figure 4** shows that inducible NOS is not effectively upregulated *in vivo* in the gut by rabbit STEC in the rabbit loop model, at least not at the 20 h time point. Our results are in agreement with those of Maresca et al. in cultured cells (Maresca et al., 2005), and in contrast to intracellular pathogens such as Salmonella. Vareille et al. have shown that heme oxygenase, an enzyme present in STEC strains but absent in laboratory *E. coli* strains, blocks the induction of NO production from host cells (Vareille et al., 2008). On the other hand, Naili et al. recently showed that NO was slowly induced during a 7 day STEC infection in mice (Naïli et al., 2020). They used a clever reporter strain construct that would irreversibly convert to sucrose-resistant if the bacteria were exposed to NO *in vivo*. After 5 days of infection, only about 50% of the bacteria showed sucrose-resistance. That study by Naili showed that the duration of infection is an important variable in assessing the role of nitric oxide. The magnitude of induction of nitric oxide by STEC was modest, however, compared to Salmonella, in which iNOS expression and NO production can increase massively *in vivo* {Barbieri, 2000 #6924}. **Figure 4** suggests that endogenously produced NO is not an effective host defense against EPEC and STEC in the short term, but does not contradict the evidence that exogenous nitric oxide donors might still be effective against these pathogens early in infection.

**Figure 5:**
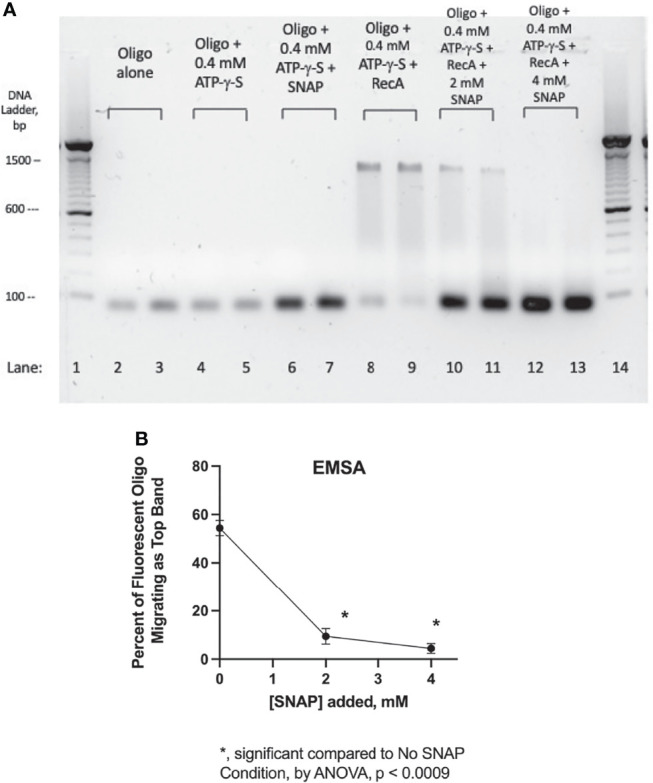
Blockade of RecA-ssDNA binding by SNAP in an electrophoretic mobility shift assay (EMSA). Panel **(A)**, a fluorescently labeled, 39-nucleotide oligonucleotide probe was used in the EMSA, which was carried out in a 1.5% agarose gel in 1 X TAE buffer plus SybrSafe dye to visualize the 100 bp markers. The image was obtained using Gel Doc EZ and “inverted” so that dark bands appear on a light background. Lanes 1 and 14 show the 100 bp DNA ladder consisting of dsDNA. Lanes 2 & 3, fluorescent oligonucleotide (“oligo”) alone. Lanes 4 & 5, oligo + adenylyl-5’-thiotriphosphate, commonly known as ATP-γ-S, a hydrolysis resistant ATP analog. In lanes 6 & 7, addition of SNAP did not alter the mobility of the oligo, although its fluorescence was enhanced. Lanes 8 & 9 show the effect of adding RecA protein, which induced a marked upward shift in the migration of the fluorescent oligo. Lanes 10-13 show the dose-dependent inhibition of the mobility shift by the addition of 2 mM and 4 mM SNAP. In this EMSA, SNAP was allowed to react with the RecA protein for 10 min before addition of the oligonucleotide. Panel **(B)**, the intensity of the bands in Panel **(A)** was quantified using Un-Scan-It gel software, and the percent of the oligo migrating as the upper band on Panel **(A)** (near the 1300 bp marker) was calculated and shown on the graph.

Lee and Singleton hypothesized several years ago that the effects of zinc on *E. coli* RecA were due to zinc’s ability to bind to amino acids Cys^90^, His^97^, and Cys^116^, which are located at the interface between adjacent RecA subunits when RecA is polymerized into a nucleofilament bound to single-stranded DNA (Lee and Singleton, 2004). We tested that hypothesis in a previous study and found that zinc acetate was able to prevent binding of RecA to a fluorescently labeled ssDNA probe (Crane et al., 2018). Nitric oxide, like zinc, can complex with cysteine residues to form nitrosothiols, which can change the biochemical properties of the target molecule or enzyme (Bauer et al., 2001; Fernhoff et al., 2009). Therefore, we tested whether the NO donor SNAP would interfere with the ability of RecA protein to bind to ssDNA in an electrophoretic mobility shift assay (EMSA). **Figure 5A** shows the effect of the NO donor SNAP on the ssDNA binding ability of RecA protein. Lanes 2 and 3 show the position of the 39-nucleotide ssDNA probe labeled with fluorescein. Addition of ATP-γ-S with or without SNAP did not change the mobility of the ssDNA probe (lanes 4-7). When RecA protein was added at a 10:1 molar ratio to the ssDNA, the mobility of the ssDNA was retarded on the gel, and ran at a position equivalent to the mobility of the 1400 bp band of the dsDNA ladder (lanes 8 & 9). A “smear” of DNA of intermediate mobility was also visible in lanes 8 -11, corresponding to the 400 to 1300 bp range of the DNA markers. When SNAP was added at 2 mM (lanes 10 & 11) or 4 mM (lanes 12 & 13), the high molecular weight bands were progressively abolished and the ssDNA probe once again ran at its original position.

**Figure 5B** shows a quantitative analysis of the band densities of the lanes 8- 13 of Panel A. For the sake of this analysis, the DNA “smears” in lanes 8-11 were ignored and only the top and bottom bands were quantitated. **Figure 5B** shows that the inhibitory effects of SNAP on formation of the high MW band were statistically significant. The EMSA analysis of **Figure 5** shows that SNAP can interfere with the ability of RecA to bind to ssDNA, in a manner similar to that of zinc. This direct effect of SNAP on RecA may be sufficient to explain the many other effects of SNAP observed on Stx production (**Figure 2**), on hypermutation (**Figure 3**), and on other aspects of the SOS response.

## Discussion

We became interested in nitric oxide as a potential inhibitor of the SOS response and Stx production based on the article by Vareille et al. (2007). On the other hand, two earlier studies indicated that a different nitric oxide donor, dinitrosyl iron, induced the SOS response rather than inhibiting it (Lobysheva et al., 1999; Stupakova et al., 2000). In the present study, we compared the effects of three different NO donors on SOS responses, including SNAP, sodium nitroprusside, and NOR-4, and tested effects both *in vitro* and *in vivo*. NO donors inhibited the induction of RecA, an early and measurable marker of the SOS response (**Figure 1**). SNAP inhibited the production of Stx toxin protein as well as abundance of *stx2* RNA (**Figure 2**). We extended the previous work mentioned above by testing if NO donors could inhibit SOS-induced hypermutation *in vitro* or *in vivo*, and found that the NO donor SNAP could inhibit hypermutation triggered by zidovudine and ciprofloxacin (**Figure 3**). We also examined if infection of ligated rabbit ileal segments with rabbit EPEC strain E22 or rabbit STEC strain E22-stx2 would trigger an effective nitric oxide production by the host, in the absence of exogenous nitric oxide donors. In this 20 h model of infection, nitric oxide metabolites in the intestine al loops did not increase during infection with strain E22, and actually decreased slightly during infection with E22-stx2 (**Figure 4**). Abundance of RNA encoding rabbit iNOS also decreased significantly during infection with E22-stx2 (**Figure 4D**). Heme oxygenase produced by STEC is one mechanism by which NO production could be suppressed (Vareille et al., 2008).

Our previous work had shown that low concentrations of zinc could block the ability of the RecA protein to bind to ssDNA, a critical early step in the induction of the SOS response (Crane et al., 2018). Since these zinc effects were thought to be mediated by zinc binding to critical cysteine residues, Cys^90^ and Cys^116^ in the RecA molecule (Lee and Singleton, 2004), we thought these two cysteines might also be “druggable” using nitric oxide donors, to form nitrosothiols. Indeed, SNAP abolished the ability of RecA to bind to a ssDNA probe in an electrophoretic mobility shift assay (**Figure 5**). If zinc and SNAP are acting upon common molecular targets, the cysteine residues in RecA, this would explain why zinc and SNAP can have additive inhibitory effects on Stx (**Figure 2**). Other authors have noted that zinc and nitric oxide can potentiate one another in bacteria (Aravindakumar et al., 1999; Binet et al., 2002; Schapiro et al., 2003). The consensus among these reports is that NO can mobilize zinc from binding sites on bacterial proteins, especially metallothionine. Therefore, the additive effects of NO and zinc observed in this study are consistent with the published literature.

Ostensibly, our results would seem to contradict the findings of Lobysheva et al., who reported that dinitrosyl iron activated, rather than inhibited, the SOS response (Lobysheva et al., 1999). A closer examination of that report, however, showed that those authors also tested SNAP as a nitric oxide donor, and found it mostly lacking in ability to activate the SOS (Figure 1 of that article). Indeed, they showed that dinitrosyl iron formed an iron-bound nitrosothiol, Fe^2+^-NO-S- R, so this may explain the apparent initial discrepancy. Dinitrosyl iron represents a unique type of nitric oxide donor, therefore, and its properties are apparently not generalizable to other NO donors. Our recent findings showed that iron, unlike zinc, could enhance SOS-induced hypermutation *in vivo* (Crane et al., 2021).

Naili et al. recently reported that endogenously produced nitric oxide, generated *via* iNOS, could apparently worsen the glomerular kidney damage in a 7 day mouse model of STEC infection (Naïli et al., 2020). Inhibition of iNOS using L-nitro-arginine methyl ester (L-NAME) protected against the glomerular injury. As stated by those authors, the effects of NO *in vivo* appear to be quite variable and highly sensitive to the environmental conditions in which it is produced, such as anaerobic vs. aerobic conditions, early vs. late in infection, and so on. In other words, nitric oxide released by exogenous NO donors early in infection could be protective, while endogenous NO produced later in infection could have detrimental effects. These late deleterious effects might not be due to *via* effects of NO on bacteria at all, but rather *via* effects on host tissues, such as increased translocation of Stx due to effects on the host intestinal mucosa {Potoka, 2002 #6925}, or increased blood flow *via* NO-induced vasodilation, etc. Our results all indicate that NO donors inhibit the SOS response in *E. coli* in general, and inhibit SOS-induced hypermutation and Stx production in EPEC and STEC in particular.

The concentrations of nitric oxide donors used in our study, as well as in the previous studies mentioned (Vareille et al., 2007), are higher than the NO concentrations generally achieved *in vivo* by endogenous production. There are many examples in biomedicine, however, where the concentrations achieved by therapeutic drugs *in vivo* are much higher than those produced endogenously. Examples would include adrenaline (epinephrine) administered to treat a cardiac arrest, or calcitriol, a potent Vitamin D analog, administered for renal osteodystrophy, where the therapeutic dose is ~ 100 times the usual amount of Vitamin D needed in a healthy person (400 IU per day). In other words, there is no law or rule stating that exogenously administered drugs must be given at doses mimicking the levels of their endogenous counterparts; what matters more is the therapeutic ratio.

The rise in multi-drug resistant bacteria has sparked concern around the world, especially in Gram-negative pathogens (Young, 2013). Our previous work showed that zinc could block the emergence of new antibiotic resistance *in vitro* in *E. coli, Klebsiella pneumoniae*, and *Enterobacter cloacae* (Bunnell et al., 2017; Crane et al., 2018), and this was confirmed by other investigators (Buberg et al., 2020). The inhibitory effect of zinc on SOS-induced antibiotic resistance was also observed *in vivo* (Crane et al., 2021). The results of the current study suggest that the combination of zinc plus a nitric oxide donor could show additive inhibitory activity against emergence of new antibiotic resistance. *In vivo*, the most logical choice of NO donors would be one of the oral nitrovasodilators, isosorbide mononitrate or isosorbide dinitrate, since these have been used in human medicine for three decades and release NO slowly in the GI tract. Targeting the GI tract makes sense since it is the anatomical site from which most multi-drug resistant Gram-negative pathogens emerge (Snitkin et al., 2012). Further work on NO donors, zinc, and the SOS response may bring beneficial insights in human and veterinary medicine for treatment and prevention of infections.

## Materials and Methods

### Bacterial Strains Used

Bacterial strains used are listed in **Table 1**. Bacteria were grown overnight in LB broth at 37°C with 300 rpm shaking, then subcultured into the medium for the expression studies, usually DMEM medium or minimal medium. In this report, when bacteria were subcultured in “DMEM” this refers to DMEM/F12 medium supplemented with 18 mM NaHCO_3_ and 25 mM HEPES, pH 7.4, but without serum or antibiotics. Addition of SOS inducers and NO donors was delayed until 1 h after initiating a subculture.

**Table 1:** *E. coli* and enteric bacterial strains used.

Strain Name	Serotype	Comment	Reference
Enteropathogenic *E. coli* (EPEC) Strains			
E22	O103:H2	Rabbit EPEC	(Milon et al., 1999; Crane and Shulgina, 2009)
Shiga-toxigenic *E.coli* (STEC) Strains, also known as Enterohemorrhagic *E. coli*			
EDL933	STEC; O157:H7	Reference Strain; stx1+ stx2+	(Perna et al., 2001)
Popeye-1	STEC; O157:H7	U.S. spinach-outbreak strain; stx2, stx2c	(CDC, 2006; Crane et al., 2011)
E22-stx2	O103: H2	Rabbit EPEC strain E22 selected for acquisition of bacteriophage 933W conferring stx2 expression	(Crane et al., 2011)
Laboratory *E. coli*, recA reporter strain			
JLM281	*recA-lacZ* reporter strain derived from laboratory strain MC4100	*recA* is used as a measure of the SOS response in *E. coli*	(Mellies et al., 2007)
MCamp	*bla-lacZ* reporter	Control reporter strain	(Mellies et al., 2007)
Non-*E. coli* Strains			
*Enterobacter cloacae* E_clo_Niagara	Not determined	Bloodstream isolate, extended spectrum ß-lactamase producer, CTX-M27.	(Crane et al., 2018)

### Materials

S-nitroso-acetyl-penicillamine (SNAP) was obtained from Cayman Chem, cat. no 82250. Sodium nitroprusside was from Sigma-Aldrich (cat. no. is no longer listed). The NO donor NOR-4, N-[(E)-4-Ethyl-3-[(Z)-hydroxyimino]-5-nitro-3-hexen-1- yl]-3- pyridinecarboxamide, was from Enzo Life Sciences. NOR-4 is an uncommonly used NO donor and it became unavailable shortly after beginning this project because the sole supplier, Dojindo Laboratories, discontinued its manufacture.

### *In Vivo* Infection of Ligated Rabbit Intestinal Loops

Rabbit experiments were performed as previously described in the Supplemental Methods of a recent article (Crane et al., 2021); fluids and tissues were collected at 20 h (Crane et al., 2007). The inoculum used to infect the loops was 4 x 10^8^ cfu per loop in 2 ml of HEPES buffered saline. Animal experiments were reviewed and approved by the IACUC of the University at Buffalo.

### Shiga Toxin Assay

Enzyme immunoassay (EIA) for Shiga toxins was performed as described (Crane et al., 2011) using the Premier EHEC toxin EIA kit (Meridian Biosciences, Cincinnati, OH). Shiga toxin 1 and Shiga toxin 2 toxoids were a kind gift of Dr. Allison Weiss, Univ. of Cincinnati, and were used to create standard curves to allow better quantitation. Since STEC strain EDL933 produces both Stx1 and Stx2, toxin production with this strain was expressed as the A_420_ values from the EIA, divided by OD_600_ to normalize for effects on growth.

### Nitric Oxide Assay

The NO metabolites nitrate and nitrite were measured using an assay kit from Cayman Chemical, cat. no. 78001. In keeping with the instructions, NO samples were passed through 10,000 MW cut-off filters (VWR or Corning Costar) to remove proteins and interfering substances before assay. Rabbit loop fluids were diluted 5-fold and rabbit serum was diluted 10-fold prior to the NO assay. In this assay, nitrate is reduced to nitrite using the enzyme nitrate reductase, and the nitrite is detected using the Griess reagent to generate a purple color and measured in a spectrophotometer.

### Miller Assay for Expression of ß-Galactosidase in Bacterial Reporter Strains

Strain JLM281, the reporter strain containing the *recA-lacZ* construct, was used to measure *recA* expression in response to inducing antibiotics, zinc and NO donors. We used a version of the Miller assay adapted to 96 well plates for higher throughput (Griffith and Wolf, 2002). However, we used 0.1% hexadecyltrimethylammonium bromide (HTA-Br) detergent alone, without chloroform or sodium dodecyl sulfate (SDS), to permeabilize the bacteria (Bunnell et al., 2017).

Briefly, we subcultured strain JLM281 at a dilution of 1:100 from an overnight culture into DMEM broth; after 1 h at 37°C, the SOS inducers or NO donors were added, and the suspension pipetted into a 96 well plate, 150 µl per well, and incubation was continued on a Bioer Mixing Block 101 thermal mixer (Bulldog Bio) at 37°C with mixing at 500 rpm. The 96 well plate was sealed with plate sealing film to prevent evaporation during the growth phase. Conditions were tested in quadruplicate. The ß-galactosidase reaction was measured using a substrate solution with 1 g/L O-nitrophenyl-ß-galactoside (ONPG) as described (Crane et al., 2021). The enzyme reaction plate was incubated at 30°C for 30 min, then A_420_ was measured on the 96 well plate reader. We omitted the addition of the Na_2_CO_3_ stop solution. Miller units were calculated using the simplified equation:


$$1000\times \frac{A_{420}}{OD_{600}\times (volume\;sampled\;[0.02\;mL])\times reaction\;time\;in\;\min\;[usually\;30]}.$$

### Expression of Bacterial Virulence Genes and Stx Toxin Assay

Quantitative RT-PCR for bacterial genes was performed as described (Crane et al., 2011) using SYBR Green as the indicator dye. The primers for *recA* and *stx2* were those used by Varielle et al. (Vareille et al., 2007) and are:

recA-for GGTAAAACCACGCTGACGTT
recA-rev ATATCGACGCCCAGTTTACG
stx2-for CACATTTACAGTGAAGGTTGA
stx2-rev TTCAGCAAATCCGGAGCCTG

### Quantitative PCR Assay for Rabbit iNOS

To measure iNOS in rabbit ileum, full-thickness pieces of intestine of ~ 40 mg in weight were collected at the end of the 20 h infection period and collected in RNAlater (Ambion, Austin, TX). This corresponds to a piece of intestinal wall about the diameter of an ordinary pencil eraser; mesenteric fat and Peyer’s patches were avoided during this collection. RNA was extracted using GeneJET RNA purification kits (Fermentas/Thermo-Fisher, Marion, OH), which performed as well as the Qiagen RNA purification kits used previously. RNA was reverse transcribed as previously reported (Crane et al., 2007) except that oligo-deoxythymidine (oligo-dT) primers were used. We tested 2 previously published PCR primer sets for rabbit iNOS but were dissatisfied with their performance because of either non-amplification of many samples or multiple peaks on melt curve analysis. Therefore we designed yet a third PCR primer pair for rabbit iNOS using PrimerQuest software at www.idtdna.com using the rabbit iNOS gene, accession number XM_002718780, as the template. The third primer set, shown here, gave acceptable results.

iNOS-forward 5’-TGAATACCAGCTGAGCAACCTGGA-3’
INOS-reverse 5’- ACCTGAACTTGTTGGTGAGCTCCT –3’

As normalizing gene we used the rabbit GAPDH primers described by Allen et al. (2004), namely, GAPDH forward 5’-TCACCATCTTCCAGGAGCGA-3’, and GAPDH reverse 5’-CACAATGCCGAAGTGGTCGT-3’.

Annealing temperature for iNOS PCR was increased to 63°based on an annealing-temperature optimization experiment. Extension was at 72 °, and melting temperature of 95°using a 3-step PCR thermal cycling protocol, for 40 cycles. PCR reactions were performed in quadruplicate.

### Electrophoretic Mobility Shift Assay (EMSA) for RecA-ssDNA Interaction

The EMSA was carried out as previously described (Crane et al., 2018) except that the purified RecA protein was from New England Biolabs instead of Abcam (NEB cat. no. M0249). The 10 X reaction buffer supplied with the RecA was used. As previously described, the ssDNA probe was a fluorescent 39-mer oligonucleotide labeled with fluorescein (IDT DNA). The 100 bp DNA ladder was from Invitrogen/ThermoFisher, cat. no. 15628-019. Adenosine 5’-(gamma-thiotriphosphate), or ATP-γ-S, was from Sigma-Aldrich, cat. no. A1388. Fluorescent bands were imaged using the Gel Doc EZ instrument from Bio-Rad and using the corresponding Image 5 software.

### Data Analysis and Statistics

Error bars shown on graphs and in Tables are standard deviations. Statistical significance was tested by ANOVA using the Dunnett’s test for multiple comparisons. For some data which was highly skewed the Kruskall-Wallis test was done instead of ordinary ANOVA. GraphPad Prism, version 9.1, was used to perform statistical analyses. Un-Scan-It Gel, from Silk Scientific, was used to quantitate bands on the agarose gels.

## Data Availability Statement

The raw data supporting the conclusions of this article will be made available by the authors, without undue reservation.

## Ethics Statement

The animal study was reviewed and approved by University at Buffalo IACUC.

## Author Contributions

All authors listed have made a substantial, direct, and intellectual contribution to the work, and approved it for publication.

## Funding

This research was supported by grant R21 AI 145836 from the National Institutes of Health, NIAID.

## Conflict of Interest

The authors declare that the research was conducted in the absence of any commercial or financial relationships that could be construed as a potential conflict of interest.

## Publisher’s Note

All claims expressed in this article are solely those of the authors and do not necessarily represent those of their affiliated organizations, or those of the publisher, the editors and the reviewers. Any product that may be evaluated in this article, or claim that may be made by its manufacturer, is not guaranteed or endorsed by the publisher.
