# Supplementary material for: Inhibition of SOS Response by Nitric Oxide Donors in Escherichia coli Blocks Toxin Production and Hypermutation
Source: Front Cell Infect Microbiol. 2021 Dec 22;11:798136. doi: 10.3389/fcimb.2021.798136 (PMC8727911; doi:10.3389/fcimb.2021.798136)
Supplement: Supplementary file 1 [file DataSheet_1.pdf]

Supplemental Figure 1

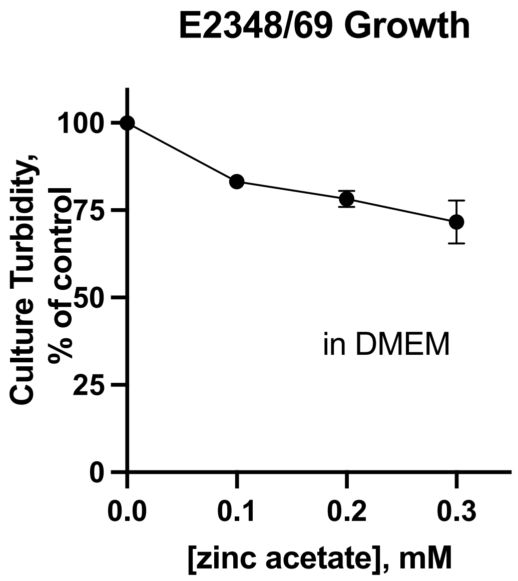

At the request of the reviewers, we include this supplemental figure showing the effect of zinc alone, without any nitric oxide donor, on growth of enteropathogenic *E. coli* strain E2348/69, as measured by OD_{600}.

Supplemental Methods for Rabbit Surgery. 2021.

The rabbit loop surgery is performed in an animal operating room using sterile gloves, sterile drape, and sterile instruments. Surgical personnel will perform a full operative "scrub" with antiseptic (chlorhexidine) soap, and will wear gowns, masks, head covers, and foot covers as well as sterile gloves as mentioned above.

A 12.5 mcg fentanyl patch is placed on the rabbit's back between the shoulder blades 24 h prior to surgery.

Ketamine and xylazine are administered as pre-anesthetics. Ketamine is up to 35 mg/kg IM, as required, and xylazine up to 5 mg/kg, as required. This injection is given according to the instructions of the LAF, namely in the posterior hind leg of the rabbit (gluteus muscle) or the anterior hind leg (quadriceps muscle).

Rabbits are given the pre-anesthetic I.M., the belly is shaved with clippers, and the rabbit is placed on a surgical table on a warming pad and the ventral abdomen is prepped with 2 % chlorhexidine in alcohol (Chloraprep). Sterile drapes are placed. The abdomen is pulled upward with a tissue clamp and a small stab incision is made in the midline; the abdominal wall continues to be retracted upward while the incision is extended with blunt scissors to approximately 6 cm in order to avoid injuring the bowel.

The intestine is exposed (usually it protrudes out of the abdominal incision spontaneously) and kept moist with gauzes moistened with warm sterile saline. The cecum and appendix are identified and used to locate the distal ileum. If there are intestinal contents in the ileum, they are milked distally or proximally using gloved fingers to empty the ileal segments.

Ileal loops about 10 cm in length are created by tying with size 0 silk sutures. Vascular arcades in the mesentery are identified and loops are created in order to preserve the blood supply. Usually seven loops are created per rabbit. We place double ties between the loops to try to prevent any leakage or cross-contamination between loops.

Into each of the loops is injected 2 ml of a suspension of E. coli or other bacteria (or sterile saline as a control) containing ~ 2 x 10^8 cfu/ml using a 26 gauge needle and small syringe. In some loops zinc or an inducing antibiotic are added along with the bacterial inoculum. The injection is done carefully along the anti-mesenteric surface of the intestine in order to avoid blood vessels, and care is taken to avoid any spillage into the peritoneal cavity. (If by accident there is inadvertent spillage into the peritoneum, the peritoneum is lavaged with sterile saline followed by lavage with 20 µg/ml gentamicin.) The intestine is returned to the abdominal cavity. The muscle layer is closed with 3/0 Vicryl using interrupted sutures. Then the skin is closed with surgical staples.

The initial phase of recovery is on the operating table with the rabbit lying on its side, still breathing supplemental oxygen, and on the warming pad. 10 to 20 mL of sterile saline are administered I.V. as fluid resuscitation. After about 15 min, the rabbit is transferred to its cage and recovery is allowed to continue. The fentanyl patch previously placed is allowed to remain in place on the skin to continue to provide analgesia. (Fentanyl patches last 48 to 72 h.) The rabbit is monitored closely (every 15 min at least) over the next hour to make sure the rabbit is

regaining consciousness. By 1 hour post-op the rabbit is able to maintain upright posture and is resting quietly in its cage.

On the 2nd day, 19 to 23 h later, the animal is given pre-anesthesia again with ketamine/xylazine. Blood may be drawn via ear vein. The rabbit is euthanized using pentobarbital (Fatal Plus) given I.V. via ear vein. Then the abdomen is re-opened and the loops are exposed. The diaphragms are punctured to ensure that death ensues. The ileal loops are removed from the rabbit's abdomen and placed on a clean blue pad and photographed. Loop fluid is collected and intestinal tissue is saved for bacteriology, Western Blot expression studies, RNA expression, assays of cytokines, and so on.
